# Progressive Heart Failure and Death as the Initial Manifestation of NK/T-Cell Lymphoma: A Case Report and Literature Review

**DOI:** 10.3389/fcvm.2021.685736

**Published:** 2021-06-24

**Authors:** Ziyu Zhang, Shuai Wang, Qingchun Liang, Daoquan Peng

**Affiliations:** ^1^Department of Cardiovascular Medicine, The Second Xiangya Hospital, Central South University, Changsha, China; ^2^Department of Pathology, The Second Xiangya Hospital, Central South Univerisity, Changsha, China

**Keywords:** NK/T-cell lymphoma, heart failure, cytokine strom, inflammation, myocarditis

## Abstract

Natural killer/T-cell (NK/T-cell) lymphoma is a rare-type non-Hodgkin lymphoma derived from NK cells or cytotoxic T cells. Here, we present a case of a 40-year-old woman who experienced quick-developed global heart failure and then was diagnosed with NK/T-cell lymphoma through lymphoid biopsy. Neither transthoracic echocardiography nor any radiological images detected a mass in her heart or pericardium. Elevated plasma troponin level and diffused patchy areas of gadolinium late enhancement on cardiac magnetic resonance were compatible with myocarditis. Considering the persistently elevated cytokine level, systemic inflammation symptoms, acute respiratory distress syndrome, and cardiac dysfunction, a cytokine storm secondary to NK/T-cell lymphoma was considered. Due to the refractory malignant arrhythmia, the patient died soon after being admitted to our hospital.

## Introduction

Natural killer (NK)/T-cell lymphoma (NKTL) is a rare and aggressive type of non-Hodgkin lymphoma derived from NK cells or cytotoxic T cells (1). NKTL mostly occurs in the nasal area and upper aerodigestive tract; although extranodal lymphoma was reported in about 30% of non-Hodgkin lymphoma (NHL) (2), cardiac NHL is rarely reported in clinical settings (3, 4).

Compared with diffuse large B-cell lymphomas and T-cell lymphomas, NKTL has a higher tendency to invade heart. Moreover, the presence of cardiac involvement is associated with poor prognosis in patients with lymphoma (5).

Cytokine storm is an umbrella term encompassing several disorders of immune dysregulation characterized by constitutional symptoms, systemic inflammation, and multiple dysfunctions that can lead to multiorgan failure (6). There are multiple clinical causes of cytokine storms, including iatrogenic, pathogen-induced, and monogenic and autoimmune disorders. Of note, hemophagocytic lymphohistiocytosis caused by Epstein–Barr virus (EBV) infection in patients with genetic susceptibility can trigger a cytokine storm (6).

Here, we report a case of an EBV-positive NKTL patient who presented progressive heart failure as an initial manifestation without evidence of cardiac lymphoma infiltration, and a lymphoma-induced cytokine storm was considered the cause of cardiac injury and rapidly deteriorating heart failure.

## Case Presentation

**Figure 1:**
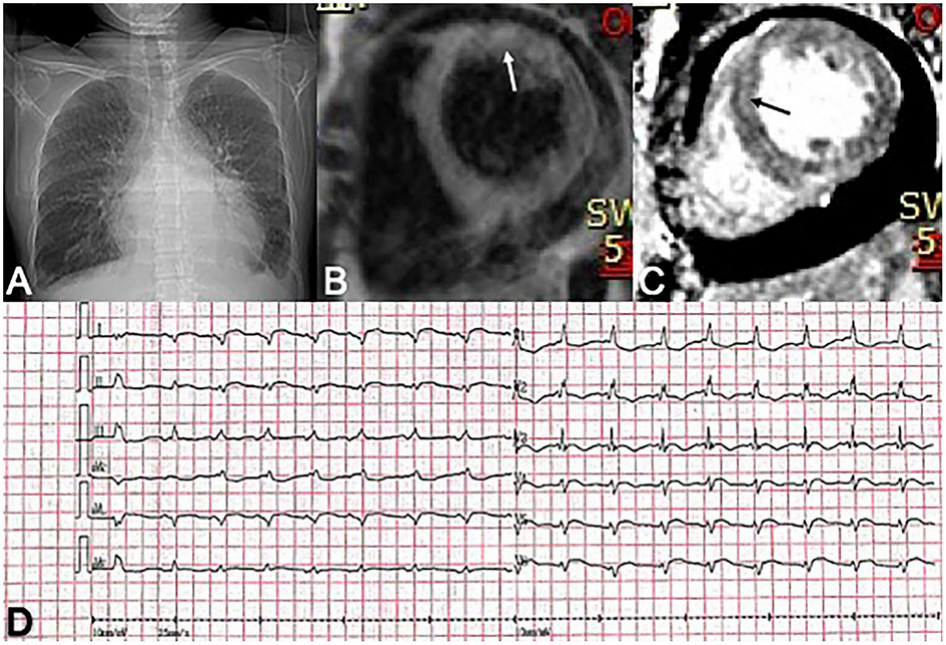
**(A)** A chest X-ray shows pleural effusion and enlarged heart shadow. **(B,C)** Diffuse patchy areas of edema (white arrow) and heterogeneous delay enhancement (black arrow) in the left ventricle on CMR images. **(D)** ECG demonstrates low voltage in all leads and ST-segment elevations of 2 mm in inferior wall leads and leads of V4, V5, and V6.

**Figure 2:**
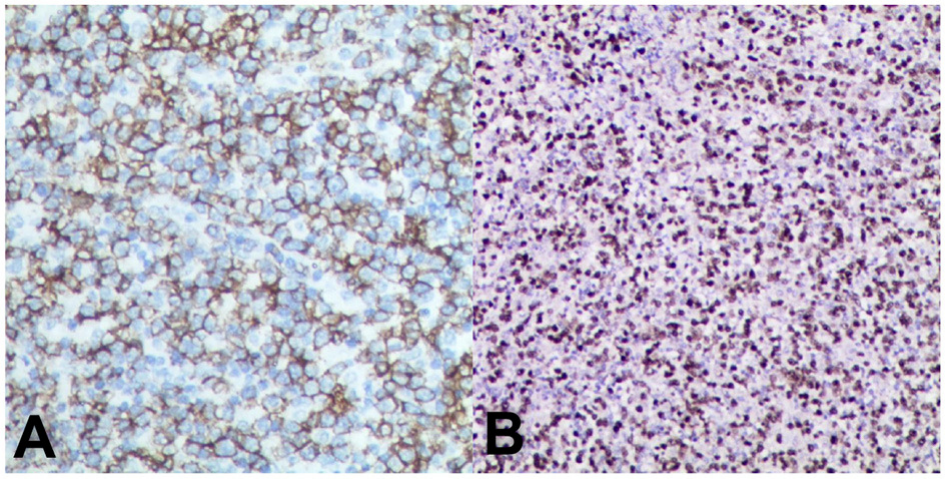
Immunochemistry of lymph nodes derived from the neck area. **(A)** Atypical cells are positive for CD56. **(B)** Cells are also positive for EBV with *in situ* hybridization.

A 40-year-old woman was admitted to our hospital because of fever and orthopnea. A month before her admission, she felt general weakness and was hospitalized. At that time, a laboratory test found increased N-terminal pro-brain natriuretic peptide (Nt-proBNP: 1,562 pg/ml), high-sensitivity troponin (hsTnT: 149 pg/ml), and DNA load of EBV (1,890 copies/ml). The 12-lead electrocardiography demonstrated low voltage in all leads and ST-segment elevation of 2 mm in inferior wall leads and leads of V4, V5, and V6 (Figure 1). A transthoracic echocardiogram showed a moderate pericardial effusion, posterior–lateral wall hypokinesis, and normal left ventricular contractility (LVEF = 55%). As acute coronary disease was considered, coronary angiography was performed and revealed normal coronary arteries with no atheromatous finding. Cardiac magnetic resonance (CMR) showed global gadolinium late enhancement and diffuse patchy areas of edema in the interventricular septum and lateral wall of the left ventricle (Figure 1). Myocarditis was presumptively diagnosed. The patient was prescribed with prednisone at 40 mg qd, but her condition did not improve and even deteriorated. When she was transferred to our hospital, she had severe heart failure (NYHA IV) and could not lay down more than a few minutes; otherwise, she would be out of breath. On admission, she was found to have a fever, several enlarged cervical lymph nodes measuring 0.5 cm, and enlargement of the liver and spleen. Lab tests revealed that NT-proBNP was more than 35,000 pg/ml and that hsTnT was 699 pg/ml. The patient had elevated IL-5 (18.16 pg/ml), IL-6 (18.16 pg/ml), IL-10 (31.10 pg/ml), and IFNγ (28.46 pg/ml). An echocardiogram showed ventricular wall hypokinesis and moderate mitral and tricuspid regurgitation, LVEF = 43% (additional files). Biopsy of the enlarged cervical lymph nodes was performed, and the histopathology showed atypical T cells with prominent hyperplasia and necrosis and lymph nodes lacking lymphoid follicles with structure destruction. A immunohistochemical study showed that these malignant cells were positive for CD4, CD3ε, CD163, and CD56 (Figure 2). EBV *in situ* hybridization was also positive (Figure 2). These features were compatible with the diagnosis of NKTL. Positron emission tomography-computed tomography (PET-CT) was not performed as the patient could not lie down.

The patient was refractory to pharmaceutical treatment for heart failure, and her condition deteriorated rapidly (7). She was transferred to the intensive care unit for circulatory and respiratory support and expired 8 days later because of cardiopulmonary failure (Table 1).

**Table 1:** Time line.

One month prior to presentation	• General weakness; BP 120/70 mmHg; SO_2_ 99% (breathing ambient air) • Nt-proBNP: 1,562 pg/ml; hsTnT: 149 pg/ml • ECG: low voltage in all leads and 2 mm in inferior wall leads and V4, V5, and V6 leads • Echocardiogram: moderate pericardial effusion, posterior–lateral wall hypokinesis, LVEF = 55% • CMR: gadolinium late enhancement and diffuse patchy areas of edema in the interventricular septum and lateral wall of the left ventricle • Treatment: sacubitril valsartan 49/51 mg bid p.o.; metoprolol 23.75 mg qd p.o.; prednisone 40 mg qd p.o. for 4 days
Three weeks prior to presentation	• Fever • Treatment: Acyclovir 250 mg q8h p.o.; amoxicillin and clavulanate potassium 1.2 g q8h p.o.
One week prior to presentation	• Dyspnea • Treatment: metoprolol 47.5 mg qd p.o.; spironolactone 20 mg qd p.o.; sacubitril valsartan 49/51 mg bid p.o.; furosemide 20 mg bid p.o.
At presentation	• Quickly aggravated dyspnea presenting as orthopnea and pulmonary edema; BP 101/75 mmHg; SO_2_ 95% (nasal cannula: 2 L/min) • Nt-proBNP: >35,000 pg/ml; hsTnT: 699 pg/ml • IL-5: 18.16 pg/ml (reference interval: 0–3.1 pg/ml); IL-6: 18.16 pg/ml (0–5.4 pg/ml); IL-10: 31.10 pg/ml (0–12.9 pg/ml)l; IFNγ: 28.46 pg/ml (0–23.1 pg/ml) • Echocardiogram: ventricular wall hypokinesis, moderate mitral and tricuspid regurgitation, LVEF = 43% • Biopsy of lymph nodes revealed NK/T-cell lymphoma • Treatment: sacubitril valsartan 49/51 mg bid p.o.; diuretics (tolvaptan 7.5 mg qd p.o.; furosemide 20 mg bid i.v.; spironolactone 20 mg qd p.o.); vasodilator (nesiritide 0.01 μg/kg/min); inotropic agent (deslanoside 0.2 mg once p.o.)
Three days later	• BP 90/50 mmHg; SO_2_ (nasal cannula: 2 L/min); hyperlactacidemia; multiple organ failure • Treatment: transferred to intensive care unit; invasive mechanical ventilation; extracorporeal membrane oxygen; intra-aortic balloon pump; continues renal replacement therapy; tolvaptan 7.5 mg qd; dezocine 5 mg CXWLBBR; deslanoside; inotropic agent (levosimendan, dobutamine, norepinephrine); antiarrhythmic (esmolol hydrochloride, amiodarone hydrochloride)
Ten days later	• Died of heart and respiratory failure

## Discussion

We reported a case of NKTL who presented with a quickly aggravated heart failure, elevated troponin, and diffuse patchy edema, and late gadolinium enhancement of the left ventricle on CMR, which suggested the diagnosis of myocarditis (8). Myocardial injury in lymphoma is uncommon. Previously, infiltration of lymphoma cells into the myocardium was considered as the cause of myocardial dysfunction. Compared with B-cell lymphoma and T-cell lymphoma, NKTL was reported to have a higher incidence of cardiac infiltration (5). In these reported cases of NKTL with cardiac involvement, evidence of lymphoma infiltration, which may manifest as a cardiac mass, abnormal thickening of myocardium, and pericardial effusion, was found by echocardiogram examination, CT/PET-CT, or cardiac MRI (Table 2) (9–14). These patients exhibited variable cardiac presentation, and all had a poor prognosis.

**Table 2:** Summarization of reported NKTL patients and our case.

**Case index**	**Age/sex**	**Race**	**Primary symptom**	**Cardiac manifestations**	**ECG**	**Echo**	**Other cardiac imaging**	**Location of biopsy**	**Clinical outcome**
1	40/F	East Asian	general weakness	myocarditis	ST-segment elevations in inferior wall leads and leads V4, V5, and V6; low voltage in all leads	LVEF = 40%	Global patchy areas of edema, linear late enhancement in the left ventricular lateral wall	Lymph node	Died
2. Shanhui et al J Clin Oncol 2011 Oct	26/M	East Asian	Fever, palpitation, general weakness	Arrhythmia	Wide QRS complex tachycardia	Hypokinetic posterolateral walls, pericardial effusion, LVEF = 41%	Cardiac mass over the left ventricular wall	Endomyocardial, lymph node	Died
3. Yiting et al Case Rep Hematol 2016 July	62/M	Caucasian	Nonspecific respiratory symptoms	Arrhythmia	Ventricular fibrillation	-	-	Autopsy	Died
4. Frank et al Asian Cardiovasc Thorac Ann 2019 Mar	38/M	Asian	Fever, substernal chest pain	Cardiac conduction block	Atrioventricular block	Right atrium mass	Cardiac mass and pericardial nodule with a maximum uptake value	Lung	Unknown
5. Lisa et al Hematol Rep 2011 Aug	54/M	Caucasian	Chest pain and dyspnea on exertion	Cardiac mass	-	Right atrial mass	Right atrial mass	Pericardiophrenic mass	Died
6. Yong-Son et al Inter Med 2014 Oct	23/M	East Asian	Abdominal pain	Myocardial hypertrophy	ST-segment elevation in the V1, V2, and V3 leads	Dilated RV and hypertrophied RV wall, pericardial effusion	Heterogeneous delayed gadolinium enhancement of the RV wall, abnormal hypermetabolic area in RV	Pancreas	Died
7. Ravindran et al Acta Oncol 2009	65/M	Asian	Difficulty in swallowing and sore throat	Cardiomyopathy	Supraventricular tachycardia and atrial fibrillation	LVEF = 25%−30%	Abnormal hypermetabolic area in RA	Nasal cavity and tonsil	Remained in remission

However, different from previous reports, there was no clinically detectable evidence of cardiac lymphoma infiltration on echocardiography and on CMR in our reported case. Elevated cTnT and diffuse patchy myocardial LGE were compatible with myocarditis (8). A cytokine storm secondary to NKTL was considered the possible mechanism leading to myocardial injury, malignant arrhythmia, and respiratory failure, which was different from previous reports.

Cytokine storms and cytokine releasing syndrome are life-threatening systemic inflammatory syndromes involving elevated levels of circulating cytokines and immune-cell hyperactivation that can be triggered by various reasons (6). For instance, the Coronavirus Disease 2019 (COVID-19) is characterized as a severe immune response caused by SARS-CoV-2 (the severe acute respiratory syndrome coronavirus 2) infection. Other causes for cytokine storms include autoinflammatory disorders, hemophagocytic lymphohistiocytosis, cancers, and monogenic disorders (6). Hematological malignancy, especially involving peripheral T cells or the NK-cell lineage, is the most common and has the worst prognosis for cytokine storm syndrome (15–17).

NK cells play a pivotal role in modulating the initial response of antigen-presenting cells and attenuate the subsequent activation of antigen-specific T cells, especially cytotoxic T lymphocytes (CTLs) (1). NK-cell dysfunction had been reported to cause the inability to terminate the inflammatory response of CTL and macrophage, ultimately leading to persistent releasing of pro-inflammatory cytokines and cytokine storms. Patients might exhibit acute systemic inflammatory symptoms and multiorgan dysfunction (18–20). Systemically elevated cytokines are known to be cardiotoxic and have the potential to result in profound myocardial injury and arrhythmia, as observed in patients with COVID-19.

## Conclusion

Our case and summarized previously reported cases in this manuscript suggest that (1) the cardiovascular injury is a significant contributor to the poor prognosis in patients with NKTL. (2) The cardiac injury in NKTL can manifest with a variety of clinical presentations such as cardiac lymphoma, ventricular arrhythmia, and cytokine-mediated myocarditis. (3) CMR and/or PET-CT are more sensitive than echocardiography in detecting cardiac injury in NKTL.

## Data Availability Statement

The original contributions presented in the study are included in the article/Supplementary Material, further inquiries can be directed to the corresponding author/s.

## Ethics Statement

Written informed consent was obtained from the individual(s) for the publication of any potentially identifiable images or data included in this article.

## Author Contributions

DP identified the case. ZZ and SW conducted the literature search and prepared the first draft of the manuscript. QL contributed to the pathological part of the study. All authors contributed to the articles and approved the submitted version.

## Conflict of Interest

The authors declare that the research was conducted in the absence of any commercial or financial relationships that could be construed as a potential conflict of interest.
